# Ginsenoside Rg1 attenuates ultraviolet B-induced glucocortisides resistance in keratinocytes via Nrf2/HDAC2 signalling

**DOI:** 10.1038/srep39336

**Published:** 2016-12-16

**Authors:** Jun Li, Dong Liu, Jinfeng Wu, Daniel Zhang, Binbin Cheng, Yani Zhang, Zifei Yin, Yuan Wang, Juan Du, Changquan Ling

**Affiliations:** 1 Department of Chinese Medicine, Changhai Hospital, Second Military Medical University, Shanghai 200433, China; 2 Department of Chinese Medicine, Lanzhou General Hospital, Gansu 730050, China; 3 Department of Dermatology, Huashan Hospital, Fudan University, Shanghai 200040, China; 4 Division of Cellular and Molecular Therapy, Department of Pediatrics, University of Florida College of Medicine, Gainesville, Florida 32611, USA; 5 E-Institute of TCM Internal Medicine, Shanghai Municipal Education Commission, Shanghai 201203, China

## Abstract

Oxidative stress, which occurs after ultraviolet (UV) radiation, usually results in Glucocorticoid (GC) resistance and the subsequent development of skin inflammation. One approach to protecting the skin against UV radiation is the use of antioxidants. The ginsenoside Rg1 is a novel natural antioxidant isolated from the medicinal plant *Panax ginseng C.A. Mey*. We demonstrated that UVB exposure exacerbated inflammation and reduced both the level of the glucocorticoid receptor (GR) and the efficacy of dexamethasone (Dex) in human keratinocytes (HaCaT cells). Pretreatment with Rg1 increased the expression of GR and restored Dex responsiveness to inflammation in UVB-irradiated HaCaT cells. Mechanistically, Rg1 rescued UVB-induced HDAC2 degradation. HDAC2 knockdown partially abolished the Rg1-induced up-regulation of GR and the enhancement of GC sensitivity. In addition, Rg1 reduced the production of reactive oxygen species (ROS), which preceded the up-regulation of HDAC2, and consequent sensitization of cells to Dex. Moreover, Rg1 treatment promoted the translocation and activation of Nrf2. Nrf2 knockdown partially abolished the Rg1-induced decrease of ROS production and increase of HDAC2. Rg1 also potentiated the anti-inflammatory effects of Dex in UVB-irradiated mouse skin. In conclusion, we demonstrated that Rg1 attenuated UVB-induced GC insensitivity. Notably, these effects were partially mediated by the Nrf2/HDAC2 pathway.

Topical glucocorticoids (GCs) are widely used to treat chronic inflammatory and autoimmune diseases of the skin^1^. However, the efficacy of glucocorticoids is affected by multiple factors, including oxidative stress, hypoxia, cytokines, changes in the cellular environment, immunomodulation and so on^2^. A number of reports have demonstrated that oxidative stress plays an important role in glucocorticoid insensitivity by inhibiting the expression and activity of histone deacetylase 2 (HDAC2)^3^,^4^. Ultraviolet B (UVB) irradiation is responsible for a variety of skin disorders by triggering the overproduction of reactive oxygen species (ROS)^5^,^6^,^7^,^8^; exposure to UVB (290–320 nm) typically aggravates skin inflammation^9^. In addition, some reports have suggested that enhanced cortisol production^10^,^11^,^12^,^13^ and reduced epidermal glucocorticoid receptor (GR) expression caused by UVB exposure might contribute to decreased GCs efficacy in chronic inflammatory diseases of the skin^13^.

The activated GC-GR complex up-regulates the expression of anti-inflammatory proteins by trans-activation and represses the expression of pro-inflammatory proteins by trans-repression by suppressing, for example, the MAPK and NF-κB pathways, which mediate the expression of inflammatory genes, including IL-1β, IL-6, IL-8 and TNF-α. Oxidative stress reduces the efficacy of GCs by modifying HDAC2 activity^14^. In patients with GC-resistant asthma, HDAC2 expression is markedly reduced in peripheral blood mononuclear cells (PBMCs) and alveolar macrophages^15^.

HDACs mediate histone deacetylation, thereby inducing chromatin condensation and transcriptional repression. HDACs also mediate the deacetylation of non-histone proteins, specifically transcription-related factors, thereby altering protein-protein interactions and DNA binding and regulating the transcriptional program^16^. HDAC2 is a critical component of the GR-corepressor complex that mediates the transrepression of NF-κB transcriptional activity by deacetylating histones in the promoters of pro-inflammatory genes^17^ and by deacetylating GR^18^.

Nuclear factor erythroid 2-related factor 2 (Nrf2) plays a key role in regulating steroid sensitivity via HDAC2 in response to inflammation in the mouse lung^19^. Nrf2 is a redox-sensitive basic leucine zipper transcription factor that induces a multitude of antioxidant genes^20^,^21^,^22^,^23^,^24^. Under normal conditions, Nrf2 is sequestered in the cytoplasm by Keap1. In response to oxidative stress, Nrf2 disassociates from the Keap1-Nrf2 complex and translocates to the nucleus, where it transcribes its target genes by binding to its cis-responsive element, referred to as the “antioxidant responsive element,” in the promoter region of target genes^25^. Nrf2 confers protection against UVB-induced inflammation and sunburn reactions in the skin^26^. A greater inflammatory response has been found in Nrf2-knockout mice compared with Nrf2 wild-type mice, indicating that Nrf2 plays a key role in protecting against UVB irradiation^27^.

Ginseng, the root of *Panax ginseng C.A. Meyer*, has been a key component of Chinese medicine for over 1000 years and is now one of the most extensively used alternative medicines worldwide. Ginsenosides are the active molecular components of ginseng^28^,^29^, and they have been widely used for their anti-aging, anti-cancer, and immunomodulatory functions^30^,^31^. In addition, ginsenosides hold promise as natural antioxidants that reduce ROS production^32^,^33^,^34^. Moreover, ginsenosides have been shown to up-regulate the GR^35^, which is responsible for GC sensitivity. Rg1 is the most abundant active ginsenoside in *P. ginseng*^36^,^37^. However, the ability of Rg1 to reverse ROS-dependent GC resistance remains unknown. In the present study, we evaluated the effects of the ginsenoside Rg1 on UVB-induced steroid resistance in human keratinocytes and determined that the mechanism occurs via the Nrf2/HDAC2 pathway.

## Results

### The ginsenoside Rg1 attenuated UVB-induced Dex insensitivity

**Figure 1:**
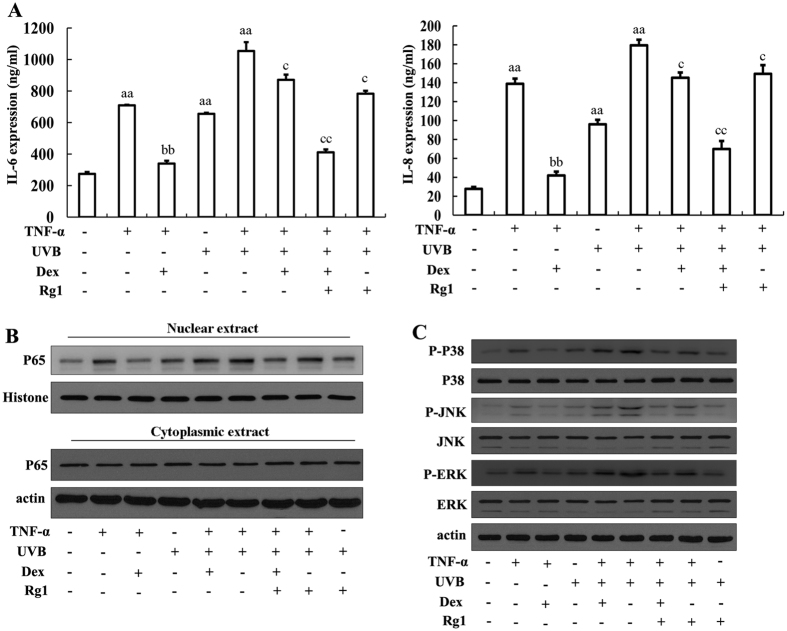
Exposure to UVB causes dexamethasone resistance in HaCaT cells. HaCaT cells were treated with dexamethasone (Dex, 1 μM) and/or Rg1 (50 μM) for 1 hour and subsequently exposed to UVB (60 mJ/cm^2^). Then, the cells were treated with 10 ng/ml TNF-α for 24 hours. (**A**) IL-6 and IL-8 levels in the cell culture supernatant were analysed using ELISA. A minimum of two independent experiments using triplicate samples provided comparable results. ^a^*p* < 0.05, ^aa^*p* < 0.01 *vs.* vehicle. ^b^*p* < 0.05, ^bb^*p* < 0.01 vs. TNF-α only. ^c^*p* < 0.05, ^cc^*p* < 0.01 vs. TNF-α+UVB. (**B**) The localization of p65 was determined using western blot analysis of nuclear and cytoplasmic protein isolates. (**C**) MAPK phosphorylation in total protein isolates was evaluated using western blot assays with anti-pP38, anti-pJNK, and anti-pERK.

First, we examined the effects of Rg1 on UVB-induced Dex insensitivity. As shown in Fig. 1A, Dex inhibited TNF-α-mediated induction of IL-6 and IL-8 in HaCaT cells. However, after UVB exposure, TNF-α-induced inflammation was enhanced, and limited inhibitory effects of Dex on IL-6 and IL-8 was observed in UVB+TNF-α+Dex group as compared with TNF-α+Dex group. Pretreatment with Rg1 significantly enhanced the anti-inflammatory effects of Dex, even after UVB irradiation. Treatment with Rg1 alone had little effect on UVB+TNF-α-induced inflammation compared with the Rg1+Dex treatment group.

In addition, Dex reduced TNF-α-induced translocation of NF-κB and the phosphorylation of MAPKs (P-p38, P-JNK and P-ERK), which mediated the secretion of IL-6 and IL-8 (Fig. 1B and C, lanes 2 and 3). However, this inhibition was attenuated after UVB irradiation (Fig. 1B and C, lanes 5 and 6). Pretreatment with Rg1 partially restored the effects of Dex on NF-κB and MAPK pathway inactivation after UVB irradiation (Fig. 1B and C, lanes 6 and 7). The quantification of the data in Fig. 1C is shown in Fig. 1SB–D.

### Rg1 up-regulated the transrepression and expression of the GR by reducing GR acetylation

**Figure 2:**
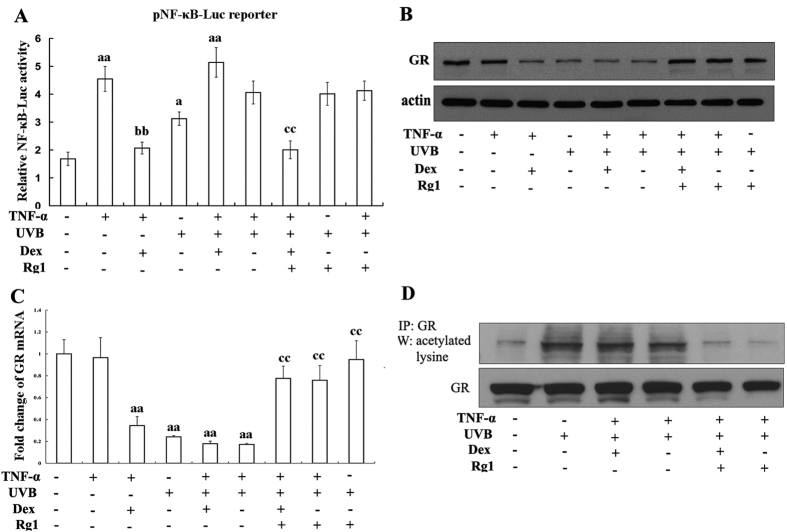
Treatment with the ginsenoside Rg1 up-regulates the transrepression and the level of GR via reducing GR acetylation. HaCaT cells were treated with Dex (1 μM) and/or Rg1 (50 μM) for 1 hour and subsequently exposed to UVB (60 mJ/cm^2^). Then, the cells were treated with 10 ng/ml TNF-α for 24 hours. (**A**) Luciferase activity in HaCaT cells transfected with pNF-κB-Luc. (**B**) GR levels were analysed using western blot assays with total protein isolates. (**C**) GR mRNA expression were determined using RT-PCR. (**D**) Acetylation of immunoprecipitated GR was evaluated using immunoblot analysis with anti-acetylated lysine and anti-GR antibodies. Each assay was repeated at least twice. ^a^*p* < 0.05, ^aa^*p* < 0.01 *vs.* vehicle. ^b^*p* < 0.05, ^bb^*p* < 0.01 vs. TNF-α only. ^c^*p* < 0.05, ^cc^*p* < 0.01 vs. TNF-α+UVB.

To understand the effects of Rg1 on UVB-induced Dex insensitivity, we next examined the GR, the GC receptor. The anti-inflammatory response mediated by the GC-GR complex occurs partially through repressing the expression of genes encoding NF-κB-dependent cytokines^18^. Therefore, we assessed GR transrepression by detecting NF-κB activation. Dex markedly reduced TNF-α-induced NF-κB-dependent luciferase activity. UVB exposure did not further increase TNF-α-induced NF-κB activation but it abolished the inhibitory effects of Dex. Pretreatment with Rg1 did not affect UVB+TNF-α-induced NF-κB activation, but it completely restored the inhibitory effects of Dex (Fig. 2A). Interestingly, GR was decreased after treatment with Dex or UVB radiation (Fig. 2B, lanes 3–6), and pretreatment with Rg1 significantly restored the level of GR in HaCaT cells after UVB irradiation (Fig. 2B; lanes 7, 8 and 9). Using realtime RT-PCR for analysis of GR mRNA, similar results were observed (Fig. 2C).

Since histone acetylation plays a significant role in the regulation of GR function^3^, we examined acetylated GR expression. As shown in Fig. 2D, UVB irradiation promoted GR acetylation, but Dex treatment had no effect. However, pretreatment with Rg1 markedly reduced GR acetylation (Fig. 2D, lanes 5 and 6).

### HDAC2 was involved in Rg1-induced GR up-regulation

**Figure 3:**
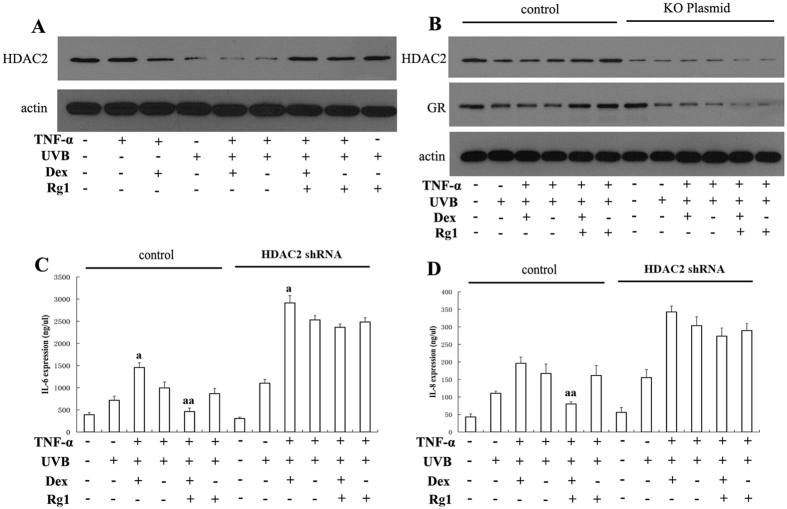
Rg1-induced HDAC2 up-regulation is essential for restoring GR levels and dexamethasone sensitivity in UVB-irradiated HaCaT cells. HaCaT cells were treated with Dex (1 μM) and/or Rg1 (50 μM) for 1 hour and subsequently exposed to UVB (60 mJ/cm^2^). Then, the cells were treated with 10 ng/ml TNF-α for 24 hours. (**A**) HDAC2 levels in total protein isolates were evaluated using western blot. (**B**) Cells were transfected with HDAC2 shRNA, and GR levels in total protein isolates were evaluated using western blot. (**C**) IL-6 levels in cells transfected with HDAC2 shRNA were assessed using ELISA. (**D**) IL-8 levels in cells transfected with HDAC2 shRNA were assessed using ELISA. ^a^*p* < 0.05, ^aa^*p* < 0.01 *vs.* TNF-α+UVB. At least two independent experiments provided highly comparable results.

HDAC2 plays a critical role in the deacetylation of non-histone proteins, specifically transcription-related factors^16^. Therefore, we investigated whether the effects of Rg1 on GR acetylation and levels were dependent on HDAC2. As shown in Fig. 3A and Fig. 2S, Dex slightly decreased HDAC2 levels, and UVB exposure significantly reduced the HDAC2 level (Fig. 3A, lanes 3–6). However, pretreatment with Rg1 partially restored UVB-induced HDAC2 degradation (Fig. 3A, lanes 7–9).

Next, we examined whether shRNA knockdown of HDAC2 could abrogate the effects of Rg1 on UVB-induced GC insensitivity. In HaCaT cells transfected with HDAC2 shRNA, the HDAC2 level was markedly reduced (Fig. 3B, top lane) and Rg1-induced up-regulation of the GR was inhibited (Fig. 3B, middle lane). Moreover, the effect of Rg1 on Dex efficacy after UVB irradiation was effectively abolished in cells transfected with HDAC2 shRNA (Fig. 3C and D) compared with cells transfected with control shRNA.

### Rg1 restored HDAC2 levels through ROS inhibition

**Figure 4:**
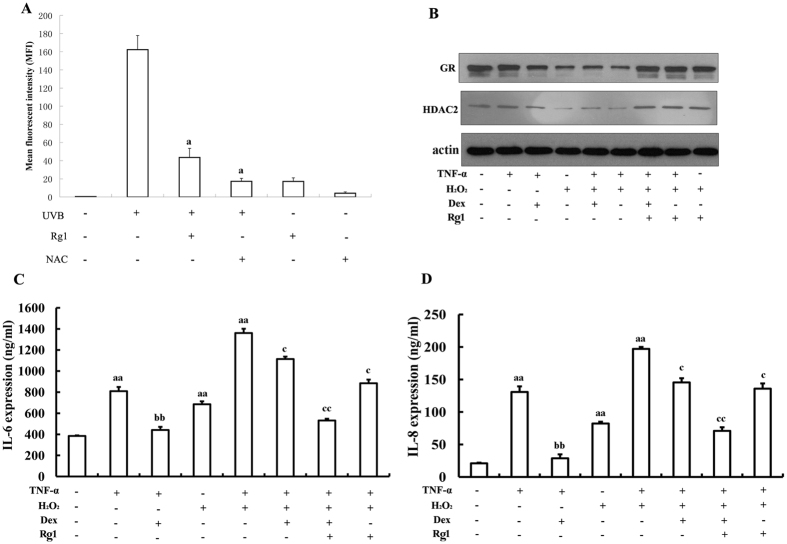
Rg1-induced up-regulation of GR and HDAC2 was mediated by the inhibition of ROS production. (**A**) HaCaT cells were pre-treated with *N*-acetyl-L-cysteine (NAC, 2 mM) or Rg1 (50 μM) for 1 hour and subsequently exposed to UVB (60 mJ/cm^2^). Fluorescence was measured using CellROX reagents. ^a^*p* < 0.01 *vs.* the UVB-irradiated group. (**B**) HaCaT cells were pre-treated with Dex (1 μM) and/or Rg1 (50 μM) for 1 hour and subsequently incubated with H_2_O_2_ for 30 minutes. Then, the cells were treated with 10 ng/ml TNF-α for 24 hours. Western blot analysis was performed using anti-GR and anti-HDAC2 antibodies. (**C**) IL-6 levels were assessed using ELISA. (**D**) IL-8 levels were assessed using ELISA. At least two independent experiments revealed highly comparable results. ^aa^*p* < 0.01 *vs.* vehicle. ^bb^*p* < 0.01 vs. TNF-α only. ^c^*p* < 0.05, ^cc^*p* < 0.01 vs. TNF-α+UVB.

Oxidative stress significantly attenuates HDAC2 activity and levels, thereby limiting the GR-mediated recruitment of GCs to DNA^38^. Therefore, we evaluated the effect of Rg1 on ROS production. As shown in Fig. 4A, UVB irradiation significantly increased ROS production. However, pretreatment with 10 μM Rg1 or 2 mM *N*-acetyl-L-cysteine (NAC, a ROS scavenger) markedly inhibited UVB-induced ROS production. Next, we determined whether the inhibition of ROS contributed to the effect of Rg1 on HDAC2 induction and GR up-regulation. As shown in Fig. 4B and Fig. 3S, hydrogen peroxide (H_2_O_2_) significantly reduced HDAC2 and GR levels (Fig. 4B, lanes 4–6). However, pretreatment with Rg1 partially reversed the effects of H_2_O_2_ on HDAC2 and GR (Fig. 4B, lanes 7–9). In addition, Rg1 potentiated the anti-inflammatory effects of Dex after H_2_O_2_ treatment (Fig. 4C,D).

### Rg1 inhibited ROS production via Nrf2 activation

**Figure 5:**
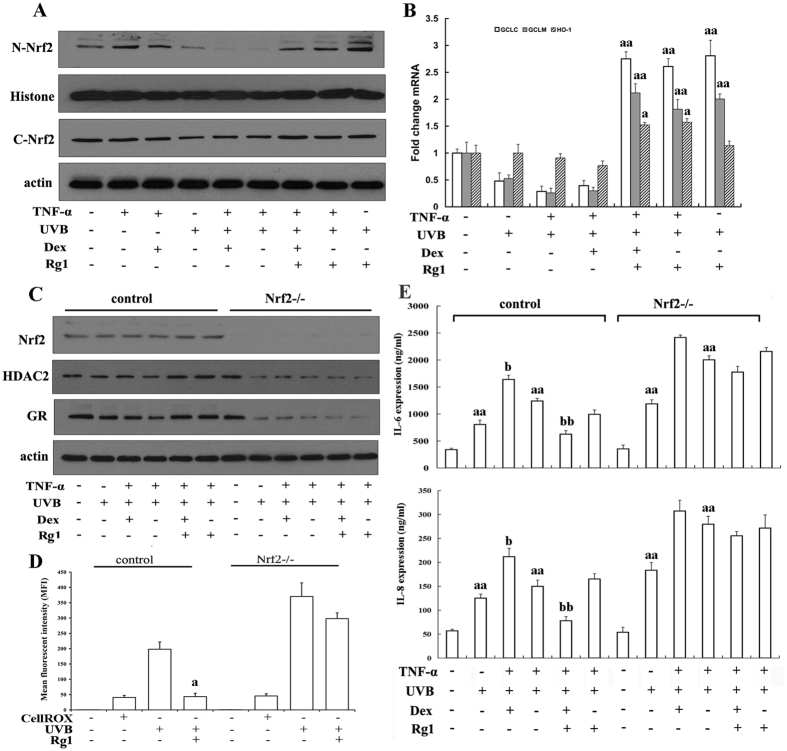
Rg1 inhibited ROS production by activating Nrf2. HaCaT cells were treated with Dex (1 μM) and/or Rg1 (50 μM) for 1 hour and subsequently exposed to UVB (60 mJ/cm^2^). Then, the cells were treated with 10 ng/ml TNF-α for 24 hours. (**A**) Nuclear and total protein were isolated as described in Materials and Methods and analysed for Nrf2, β-actin, and histone levels using western blot. (**B**) Nrf2-induced expression of HO-1, GCLC and GCLM was analysed using RT-PCR. ^a^*p* < 0.05, ^aa^*p* < 0.01 *vs.* UVB+TNF-α group. (**C**) The cells were transiently transfected with Nrf2 small interfering RNAs (siRNAs), and GR and HDAC2 levels in total proteins isolates were evaluated using western blot. (**D**) ROS in cells transfected with Nrf2 siRNA were assessed using CellROX. ^a^*p* < 0.05 *vs.* the UVB-irradiated group. (**E**) IL-6 (top) and IL-8 (bottom) levels in cells transfected with Nrf2 siRNA were assessed using ELISA. ^aa^*p* < 0.01 vs. control group; ^b^*p* < 0.05, ^bb^*p* < 0.01 *vs.* UVB+TNF-α group. (**A**–**D**) At least two independent experiments revealed highly comparable results.

A previous study demonstrated that Nrf2 increases the level of HDAC2 and plays key roles in protecting against UVB radiation and inflammation^19^. Therefore, we investigated whether Rg1 could enhance Nrf2 activity. As shown in Fig. 5A and Fig. 4S, the nuclear translocation and cytoplasmic expression of Nrf2 were reduced significantly after UVB irradiation (lanes 4 and 6). Dex treatment had no effects on this UVB-associated reduction in Nrf2 levels (lane 5), whereas Rg1 pretreatment markedly increased the nuclear translocation and cytoplasmic expression of Nrf2 after UVB irradiation (lanes 7–9). We also observed that TNF-α stimulated the nuclear translocation of Nrf2 (lane 2). RT-PCR demonstrated that pretreatment with Rg1 significantly increased the expression of Nrf2 target genes, including haem oxygenase-1 (HO-1), glutamate-cysteine ligase catalytic subunit (GCLC) and glutamate-cysteine ligase modifier subunit (GCLM)) (Fig. 5B). Knock-down of Nrf2 by siRNA markedly inhibited the Rg1-induced up-regulation of HDAC2 and the GR (Fig. 5C) and abolished Rg1-mediated inhibition of ROS production (Fig. 5D). In addition, the ability of Rg1 to promote the effect of Dex on anti-inflammatory cytokines was abolished in cells transfected with Nrf2 siRNA (Fig. 5E top and bottom), compared with cells transfected with control siRNA.

### Rg1 potentiated GC efficacy after UVB irradiation *in vivo*

**Figure 6:**
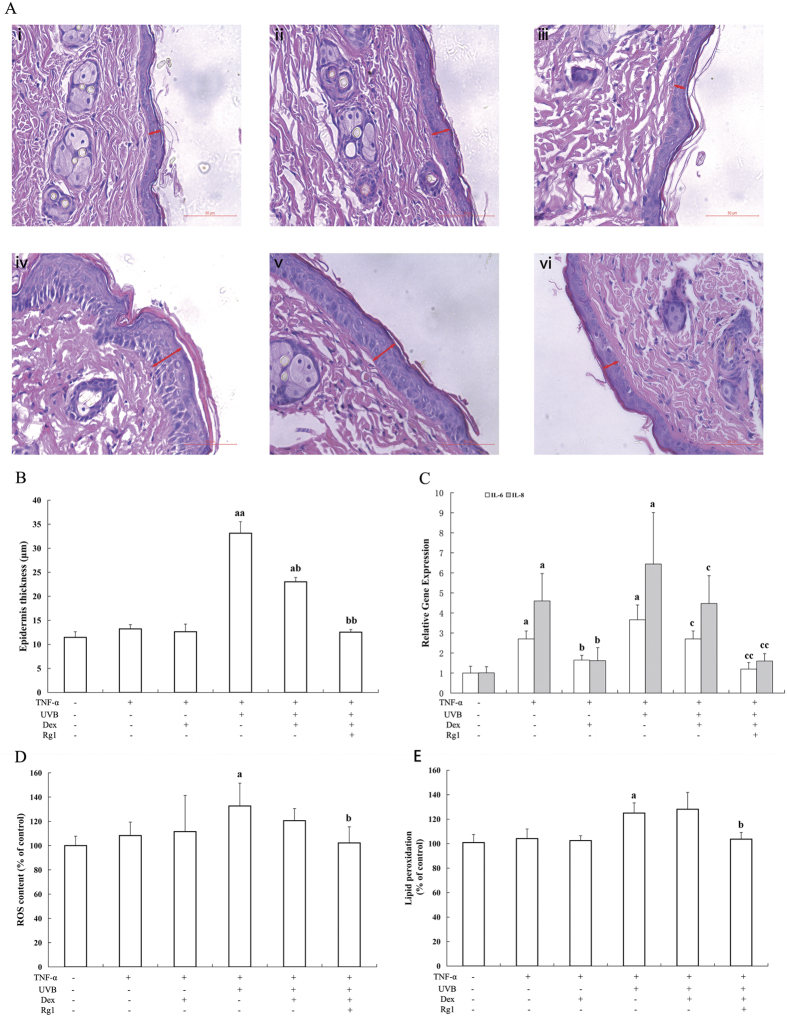
The ginsenoside Rg1 attenuated UVB-induced changes in epidermal thickness, ROS and inflammation in shaved mice. (**A**-i) control, (**A**-ii) TNF-α treatment, (**A**-iii) pretreatment with Dex prior to TNF-α; (**A**-iv) TNF-α treatment after UVB irradiation; (**A**-v) pretreatment with Dex before UVB+TNF-α; and (**A**-vi) pretreatment with Dex+Rg1 before UVB+TNF-α. (**B**) Epidermal thickness measurements were presented as the mean ± SD (*n* = 6). ^a^*p* < 0.05, ^aa^*p* < 0.01 vs. control group; ^b^*p* < 0.05, ^bb^*p* < 0.01 *vs.* UVB+TNF-α group. Expression of the pro-inflammatory cytokines in skin tissue: (**C**) IL-6 and IL-8. The values of relative mRNA expression were expressed as the mean ± SD, *n* = 6; ^a^*p* < 0.05 vs. control group; ^b^*p* < 0.05, *vs.* TNF-α group; ^c^*p* < 0.05, ^cc^*p* < 0.01 *vs.* UVB+TNF-α group. The index of the oxidative stress in skin tissue: (**D**) ROS content; and (**E**) lipid peroxidation. The values are expressed as the mean ± SD (*n* = 6). ^a^*p* < 0.05 vs. control. ^b^*p* < 0.05 *vs.* UVB+TNF-α group.

The morphology and histopathological changes of the mouse skin in the various groups are shown in Fig. 6. The red arrows in Fig. 6A(i–vi) indicate the epidermal thickness in shaved mice. The epidermal thickness in the UVB irradiation group (Fig. 6A-iv, red arrow) was clearly greater than those of the vehicle control and TNF-α treatment alone groups (Fig. 6A-i,ii). Dex treatment slightly reduced the epidermal thickness (Fig. 6A-v), and Rg1 treatment markedly decreased the epidermal thickness after UVB irradiation (Fig. 6A-vi). Furthermore, as shown in Fig. 6B, the epidermal thickness of the UVB-irradiated group (33.13 ± 2.41 μm) increased approximately 3-fold compared with the control group (11.46 ± 1.17 μm). The epidermal thickness of the Dex group (23.02 ± 0.92 μm) and the Rg1 + Dex (12.55 ± 0.57 μm) group was significantly lower than that of UVB radiation alone group.

Next, as shown in Fig. 6C, TNF-α treatment caused significant increases in IL-6 and IL-8 levels compared with the vehicle control group, and UVB irradiation enhanced this inflammatory response. Dex significantly inhibited the production of inflammatory cytokines, and the anti-inflammatory effects of Dex were reduced after UVB irradiation. Rg1 combined with Dex markedly reduced the IL-6 and IL-8 levels in skin homogenates compared with Dex treatment alone.

Oxidative stress in the form of ROS overproduction and lipid peroxidation causes skin injury in UVB irradiation-induced photodamage in shaved mice^39^. In our study, ROS levels (Fig. 6D) and lipid peroxidation (Fig. 6E) increased in the skin of UVB-irradiated mice compared with the control mice. Treatment with Rg1 in combination with Dex significantly reduced the ROS level and lipid peroxidation of the skin compared with Dex treatment alone.

## Discussion

Therapeutic strategies to restore GR levels are urgently needed to overcome the challenge of GC resistance^2^,^40^. In this study, we demonstrated that the ginsenoside Rg1 effectively reversed UVB-induced Dex insensitivity by up-regulating the GR and that these effects were partially mediated by the Nrf2/HDAC2 pathway.

Consistent with previous studies^41^,^42^, the cytotoxic effects of UVB radiation on HaCaT cells occurred in a dose- and time-dependent manner. In this study, 60 mJ/cm^2^ UVB irradiation caused a significant increase in ROS production and induced minor cytotoxic effects (Fig. 1SA); therefore, this dose was used in the remaining experiments.

We demonstrated that Rg1 enhanced the anti-inflammatory effects of Dex on UVB-irradiated cells. Rg1 treatment alone partially inhibited TNF-α-induced IL-6 and IL-8 secretion after UVB exposure. This effect was likely due to the anti-inflammatory effect of Rg1^43^, as Rg1 inhibited MAPK phosphorylation and NF-kB translocation. However, the precise mechanism of the Rg1-mediated reduction of inflammation requires further investigation. It has been reported that a defect in GR deacetylation caused by reduced HDAC2 resulted in GC insensitivity in terms of NF-κB-mediated gene expression^18^. Consistent with this finding, we found that UVB irradiation impaired Dex-mediated inhibition of NF-κB activity and that pretreatment with Rg1 blocked this effect. This phenomenon may be explained by (i) increased Rg1-mediated GR transcriptional repression or (ii) reduced Rg1-mediated GR deacetylation via HDAC2 up-regulation. A reduction in GR levels is one of the mechanisms that contributes to GC resistance^2^. We also found that Rg1 up-regulated GR expression, which might be explained by the effects of deacetylation on ubiquitin and protein binding, resulting in the loss of selective GR degradation^12^,^44^.

HDAC2 is a critical component of the GR-corepressor complex that mediates the transrepression of NF-κB transcriptional activity by deacetylating histones in the promoters of genes encoding pro-inflammatory factors^17^, and the GR^18^. Our results clearly demonstrated that HDAC2 silencing markedly inhibited, but did not fully block, the Rg1-induced restoration of GC efficacy. This phenomenon might be explained by (i) incomplete knockdown of HDAC2 at the protein level or (ii) another mechanism associated with the effect of GCs on inflammation. The level and transcriptional repression of the GR are not the only factors associated with GC sensitivity. For example, (i) GCs show non-genomic anti-inflammatory action by binding with or without the GR to activate annexin-1^45^,^46^, (ii) GCs induce the expression of many anti-inflammatory genes by transactivation of the GR^47^ or (iii) GCs activate anti-inflammatory genes by recruiting coactivator molecules such as steroid receptor coactivator 1 (SRC1), SRC2, and SRC3^48^. In this respect, the effects of Rg1 on non-genomic effects of GC, GR transactivation, and the GR-corepressor complex should be considered and further investigated in the future.

ROS reduce the efficacy of GCs by modifying HDAC2 activity^14^. We found that Rg1 inhibited UVB-induced ROS production. In addition, Rg1 rescued H_2_O_2_-induced down-regulation of HDAC2 and GC insensitivity, indicating that Rg1 restored GC responsiveness by inhibiting the effects of oxidative stress. To the best of our knowledge, Nrf2-deficient cells are sensitive to oxidative stress^25^,^49^. In our study, Rg1 treatment activated Nrf2 by increasing the translocation of Nrf2 and expression of the major Nrf2 target genes HO-1, GCLC and GCLM, which exert potent antioxidant effects in multiple cell types, including keratinocytes^50^,^51^. More importantly, Nrf2 knockdown partially abolished the inhibitory effect of Rg1 on ROS production, indicating that the effect of Rg1 on ROS production was at least partially Nrf2-dependent. Additional mechanisms, including PI3K/AKT activation, also contributed to Rg1-mediated ROS inhibition (Fig. 5S). Yasuo *et al*.^52^ reported that corticosteroid insensitivity in COPD could be reversed by inhibiting oxidative stress-dependent PI3K-δ activation. In this respect, a PI3K inhibitor or siRNA should be used to validate the contribution of PI3K/AKT to Rg1-mediated inhibition of ROS production in the future.

Furthermore, animal studies were performed to explore the combined effects of Rg1 and Dex, with the goal of identifying a safe and effective strategy to treat skin inflammation. No obvious liver or kidney damage was observed after treatment with Rg1 combined with Dex (Fig. 6S). In the present study, epidermal thickness and inflammation were used as the primary markers of UVB irradiation-induced damage. Our results indicated that Rg1 combined with Dex effectively decreased the inflammation and changes in epidermal thickness resulting from UVB irradiation. ROS overproduction and lipid peroxidation can cause skin injury in UVB-exposed mice^33^. Our results demonstrated that Rg1 effectively reduced the amount of ROS and lipid peroxidation, suggesting that Rg1 enhances the anti-inflammatory effects of Dex *in vivo* by exerting antioxidant effects.

In conclusion, the ginsenoside Rg1 attenuated UVB-induced GC insensitivity. Notably, these effects were mediated, at least in part, via the Nrf2/HDAC2 pathway. However, it is still unknown if Rg1 affects endogenous cortisol production, and the question of whether other Rg1-mediated mechanisms are involved in GC sensitivity merits further investigation. Rg1 might be a potential candidate drug to attenuate UVB irradiation-induced GC resistance in chronic inflammatory or autoimmune diseases of the skin.

## Materials and Methods

### Cell culture

The HaCaT human skin keratinocyte cell line was purchased from the American Type Culture Collection (Manassas, VA, USA). The cells were maintained in Dulbecco’s Modified Eagle’s Medium (DMEM) (Invitrogen, Carlsbad, CA, USA) supplemented with 10% FBS, 100 U/ml penicillin, and 0.1 mg/ml streptomycin at 37 °C in a humidified incubator with 5% CO_2_.

### Cytokines and reagents

Recombinant murine TNF-α was purchased from R&D (Minneapolis, MN, USA). Dex was purchased from Sigma-Aldrich (St. Louis, MO, USA). Rg1 was purchased from the National Institutes for Food and Drug Control (Beijing, China). Antibodies against the GR, acetylated lysine, NF-κBp65, phospho-MAPKs, MAPKs, HDAC2, and Nrf2 were purchased from Cell Signaling (Beverly, MA, USA).

### UVB-induced Dex resistance *in vitro*

Cells were seeded in 6- or 96-wells plates overnight. Then, the cells were pre-treated with Dex (1 × 10^−6^ M) and/or Rg1 (5 × 10^−5^ M) for 1 hour, and the medium was discarded. The cells were irradiated using a Philips TL-D/08 UVB instrument (wavelength 290–340 nm, peak 311 nm, Philips, Amsterdam, the Netherlands) with a total energy dose of 60 mJ/cm^2^. After that, the medium was added back to the cells as described. One hour later, TNF-α (10 ng/ml) was added to the wells for 24 hours.

### Measurement of reactive oxygen species

The cells were plated in black 96-well plates and allowed to attach to the wells for 24 hours. The cells were pre-treated with NAC (2 mM) or Rg1 (5 × 10^−5^ M) for 1 hour. Then, the medium was discarded and the cells were irradiated with a total energy dose of 60 mJ/cm^2^ using a UVB instrument. The cells were subsequently stained with 5 μM CellROX™ Green Reagent (Thermo Fisher Scientific, MA, USA) and incubated at 37 °C for 30 min^53^.

### ELISA

IL-6 and IL-8 levels were determined using ELISA kits according to the manufacturer’s instructions (Westang Bio-Tec, Shanghai, China).

### Western blot analysis

Whole cell protein and nuclear lysates were prepared, and the lysates were analysed using western blot assays as previously described^43^.

### Real-time PCR

The RT-PCR assays were conducted as previously described^54^.

Primers:

hGR-FW: CATCCACTGCTGTGTCTGCT

hGR-RV: GGGACCCAGAAGAAAACTCC

hHO-1-FW: ATGACACCAAGGACCAGAGC

hHO-1-RV: GTAAGGACCCATCGGAGAAGC

hGCLC-FW: AAGCCATTCACTCCAGATTTTACC

hGCLC-RV: ACAACAAACTTCAACGCAAAGC

hGCLM-FW: ACTGACTTAGGAGCATAACTTACC

hGCLM-RV: AAGAATATCTGCCTCAATGACACC

hβ-Actin-FW: CGAGGCCCAGAGCAAGAG

hβ-Actin-RV: CCACACGCAGCTCATTGTA

mIL-6-FW: GCCTTCTTGGGACTGATGCT

mIL-6-RV: TGGAAATTGGGGTAGGAAGGAC

mIL-8-FW: AGATACCGCCACGTTCTGAC

mIL-8-RV: GAAATGGAGAGGCATCCGGT

mβ-Actin-FW: CCTCTATGCCAACACAGTGC

mβ-Actin-RV: GTACTCCTGCTTGCTGATCC

### Cell transfection with siRNA and plasmid

HDAC2 and Nrf2 were silenced using the HDAC2 CRISPR/CAS 9 KO plasmid or siRNA (Santa Cruz, CA, USA) with polyethylenimine (Polyscience, IL, USA). The effect of gene silencing was evaluated using western blot analysis.

### UVB-induced skin damage

Six-week-old male Balb/c mice weighing 18–22 g were purchased from the Second Military Medical University Animal Center (Shanghai, China). The UVB-induced skin damage method was conducted as described by Chiang-Wen Lee *et al*. with some modifications^39^. The mice were randomly divided into six groups of eight mice each. Group 1, which served as the control group, was treated with PBS and did not undergo UVB irradiation. Group 2 was treated with TNF-α and did not undergo UVB irradiation. Group 3 was treated with TNF-α and Dex, and did not undergo UVB irradiation. Group 4 was treated with TNF-α and underwent UVB irradiation. Group 5 was treated with TNF-α and Dex, and underwent UVB irradiation. Group 6 was treated with Rg1, TNF-α, and Dex, and underwent UVB irradiation.

TNF-α (2.5 mg/kg) was subcutaneously injected at a dose of 100 μl per mouse after the final UVB irradiation. Dex (2.5 mg/kg) was intraperitoneally injected at a dose of 100 μl per mouse before the TNF-α treatment. Rg1 (15 mg/kg) was intraperitoneally injected at a dose of 100 μl per mouse daily for 3 weeks. The UVB source (311 nm) of simulated solar irradiation was provided by a UVB ultraviolet instrument (Sigma, SH4 B, Shanghai, China). The mice in the UVB-irradiated groups were exposed to UVB radiation three times per week for 3 weeks for a total energy dose of 600 mJ/cm^2^. A dose of 50 mJ/cm^2^ was administered during the first week, and a dose of 75 mJ/cm^2^ was administered in the remaining 2 weeks. The animals were sacrificed 24 hours after TNF-α treatment, and dorsal skin samples were immediately excised. The samples were washed with ice-cold PBS and stored at −80 °C. The skin samples were assessed for histological, antioxidant and anti-inflammatory properties. The study was conducted in accordance with the European Communities Council Directive of November 24, 1986 (86/609 EEC) and was approved by the Ethics Committee of Changhai Hospital.

### Evaluation of Lipid Peroxidation *in vivo*

Lipid peroxidation in skin tissue was evaluated as previously described by Chiang-Wen Lee *et al*.^55^.

### ROS levels *in vivo*

ROS levels in the skin tissue of mice in each experimental group were measured as previously described in ref. 56.

### Histology

The skin tissue was dissected post-mortem, fixed in 10% formalin saline solution, decalcified, dehydrated, and embedded in paraffin. Sections of 5 μm were prepared and stained with haematoxylin and eosin. The epidermal thickness of each sample was determined using a microscope.

### Statistical analysis

All of the data are presented as the mean ± standard deviation (SD). The statistical significance of the differences was determined using SPSS 11.0 for Windows (SPSS Inc. Chicago, IL, USA). The data were analysed using one-way analysis of variance (ANOVA) followed by Fisher’s least significant difference (LSD) test. Differences with *p*-values < 0.05 were considered statistically significant.

## Additional Information

**How to cite this article**: Li, J. *et al*. Ginsenoside Rg1 attenuates ultraviolet B-induced glucocortisides resistance in keratinocytes via Nrf2/HDAC2 signalling. *Sci. Rep.* **6**, 39336; doi: 10.1038/srep39336 (2016).

**Publisher’s note:** Springer Nature remains neutral with regard to jurisdictional claims in published maps and institutional affiliations.

## Supplementary Material

Supplementary Information
