# Supplementary material for: Ginsenoside Rg1 attenuates ultraviolet B-induced glucocortisides resistance in keratinocytes via Nrf2/HDAC2 signalling
Source: Sci Rep. 2016 Dec 16;6:39336. doi: 10.1038/srep39336 (PMC5159887; doi:10.1038/srep39336)
Supplement: Supplementary Information [file srep39336-s1.pdf]

Ginsenoside Rg1 attenuates ultraviolet B-induced glucocortisides resistance in keratinocytes via Nrf2/HDAC2 signalling

Jun Li[#1,2], Dong Liu[#1], Jinfeng Wu[#3], Daniel Zhang[4], Binbin Cheng[1], Yani Zhang[1], Zifei Yin[1], Yuan Wang[1], Juan Du*[1], Changquan Ling*[1,5]

[1]Department of Chinese Medicine, Changhai Hospital, Second Military Medical University, Shanghai 200433, China; [2]Department of Chinese Medicine, Lanzhou General Hospital, Gansu 730050, China; [3]Department of Dermatology, Huashan Hospital, Fudan University, Shanghai 200040, China; [4]Division of Cellular and Molecular Therapy, Department of Pediatrics, University of Florida College of Medicine, Gainesville, Florida 32611, USA. [5]E-Institute of TCM Internal Medicine, Shanghai Municipal Education Commission, Shanghai 201203, China.

*Correspondence to: Dr. Juan Du, E-mail: dujuan714@163.com. Tel: +862131161966, Dr. Changquan Ling, E-mail: lingchangquan@hotmail.com. Tel: +862181871551

[#] Contributed equally to this work

Fig. 1S. The effect of Rg1 on HaCat cells after UVB irradiation. HaCaT cells were treated with dexamethasone (Dex, 1 μM) and/or Rg1 (50 μM) for 1 hour and subsequently exposed to UVB (60 mJ/cm2). Then, the cells were treated with 10 ng/ml TNF-α for 24 hours. (**A**) Cell viability was assessed by the MTT assay. Rg1 has no cytotoxic effects on HaCat cells. (**B-D**) Band intensities of Fig. 1C were quantified using UN-SCAN-IT gel analysis software version 6 (Silk Scientific, Inc., Orem, UT, USA). The optical density for target protein was shown as a proportion of β-actin optical density. $^{a}p < 0.01$ *vs.* vehicle, $^{b}p < 0.05$, $^{bb}p < 0.01$ *vs.* UVB+TNF-α.

Fig. 2S. The effect of Rg1 on HDAC2 after UVB irradiation. Band intensities of Fig. 3A were quantified using UN-SCAN-IT gel analysis software version 6 (Silk Scientific, Inc., Orem, UT, USA). The optical density for HDAC2 was shown as a proportion of β-actin optical density. $^{a}p < 0.01$ *vs.* vehicle, $^{b}p < 0.01$ *vs.* UVB+TNF-α.

Fig. 3S. Rg1-induced GR and HDAC2 up-regulation were mediated by inhibition of ROS. Band intensities of Fig. 4B were quantified using UN-SCAN-IT gel analysis software version 6 (Silk Scientific, Inc., Orem, UT, USA). The optical density for target protein was shown as a proportion of β-actin optical density. $^{a}p < 0.05$, $^{aa}p < 0.01$ *vs.* vehicle, $^{b}p < 0.01$ *vs.* UVB+TNF-α group.

Fig. 4S. The effect of Rg1 on Nrf2 after UVB irradiation. Band intensities of Fig. 5A were quantified using UN-SCAN-IT gel analysis software version 6 (Silk Scientific, Inc., Orem, UT, USA). The optical density for N-Nrf2 and C-Nrf2 were shown as a proportion of Histone and β-actin optical density. $^{a}p < 0.05$, $^{aa}p < 0.01$ *vs.* vehicle, $^{b}p < 0.05$, $^{bb}p < 0.01$ *vs.* UVB+TNF-α.

Fig. 5S. Ginsenoside Rg1 activates PI3K/AKT in UVB-irradiated Hacat cells. After being exposed to Dex (1 μM) combined with/without Rg1 (50 μM) for 1 hour, HaCat cells were exposed to UVB (60 mJ/cm^2), then to 10 ng/ml TNFα for 24 hours. Expression of PI3K, P-AKT and total AKT, as assessed by Western-blot.

Fig. 6S. Ginsenoside Rg1 has no liver and kidney damage in mice. Hematoxylin and eosin staining of the kidney (**A**) and liver (**B**) in mice after Rg1 treatment. (i) control; (ii) TNF-α treatment; (iii) pretreatment with Dex before TNF-α; (ⅳ) TNF-α treatment after UVB irradiation; (v) pretreatment with Dex before UVB＋TNF-α; (vi) pretreatment with Dex＋Rg1 before UVB＋TNF-α.

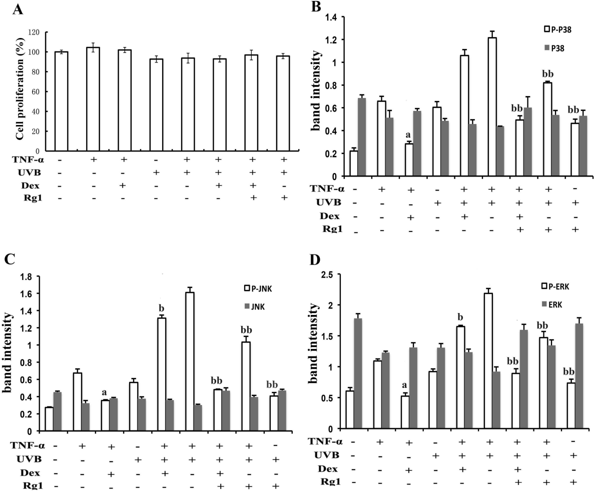
A
Cell proliferation (%)
TNF-α
UVB
Dex
Rg1
B
band intensity
P-P38
P38
a
bb
C
band intensity
P-JNK
JNK
a
b
bb
D
band intensity
P-ERK
ERK
a
b
bb

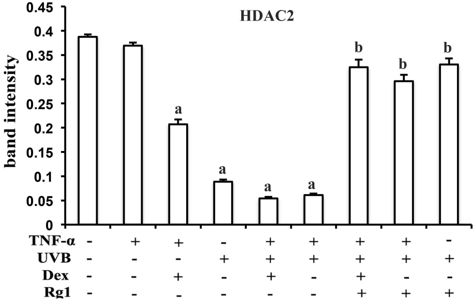

HDAC2
band intensity
0.45
0.4
0.35
0.3
0.25
0.2
0.15
0.1
0.05
0
a
a
a
a
b
b
b
TNF-α - + + - + + + + -
UVB - - - + + + + + +
Dex - - + - + - + - -
Rg1 - - - - - - + + +

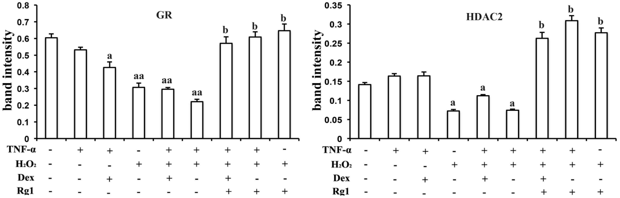

GR
HDAC2
band intensity
a
aa
b
TNF-α
H₂O₂
Dex
Rg1

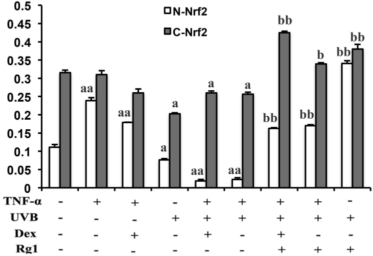

0.5
0.45
0.4
0.35
0.3
0.25
0.2
0.15
0.1
0.05
0
N-Nrf2
C-Nrf2
aa
aa
a
a
aa
a
aa
a
bb
bb
bb
b
bb
bb
TNF-α - + + - + + + + -
UVB - - - + + + + + +
Dex - - + - + - + - -
Rg1 - - - - - - + + +

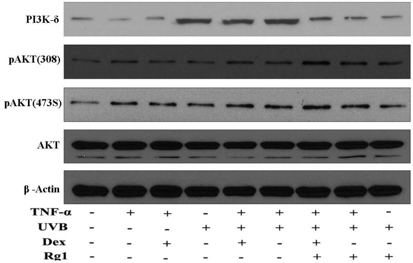
PI3K-δ
pAKT(308)
pAKT(473S)
AKT
β -Actin
TNF-α - + + - + + + + -
UVB - - - + + + + + +
Dex - - + - + - + - -
Rg1 - - - - - - + + +

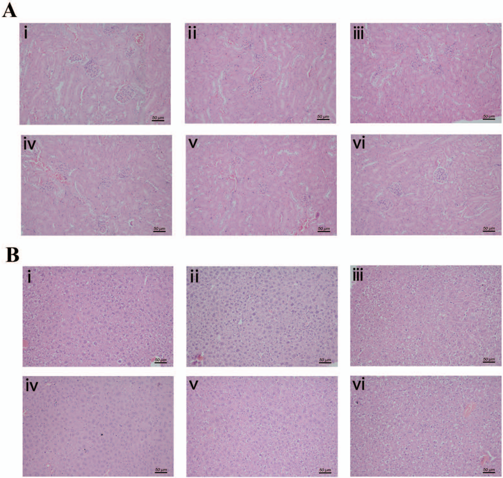
A
i
ii
iii
iv
v
vi
50 μm
B
i
ii
iii
iv
v
vi
50 μm
